# Supplementary figures and images for: Influenza A virus induces PI4P production at the endoplasmic reticulum in an ATG16L1-dependent manner to promote the egress of viral ribonucleoproteins
Source: PLoS Biol. 2025 Jul 16;23(7):e3002958. doi: 10.1371/journal.pbio.3002958 (PMC12286409; doi:10.1371/journal.pbio.3002958)

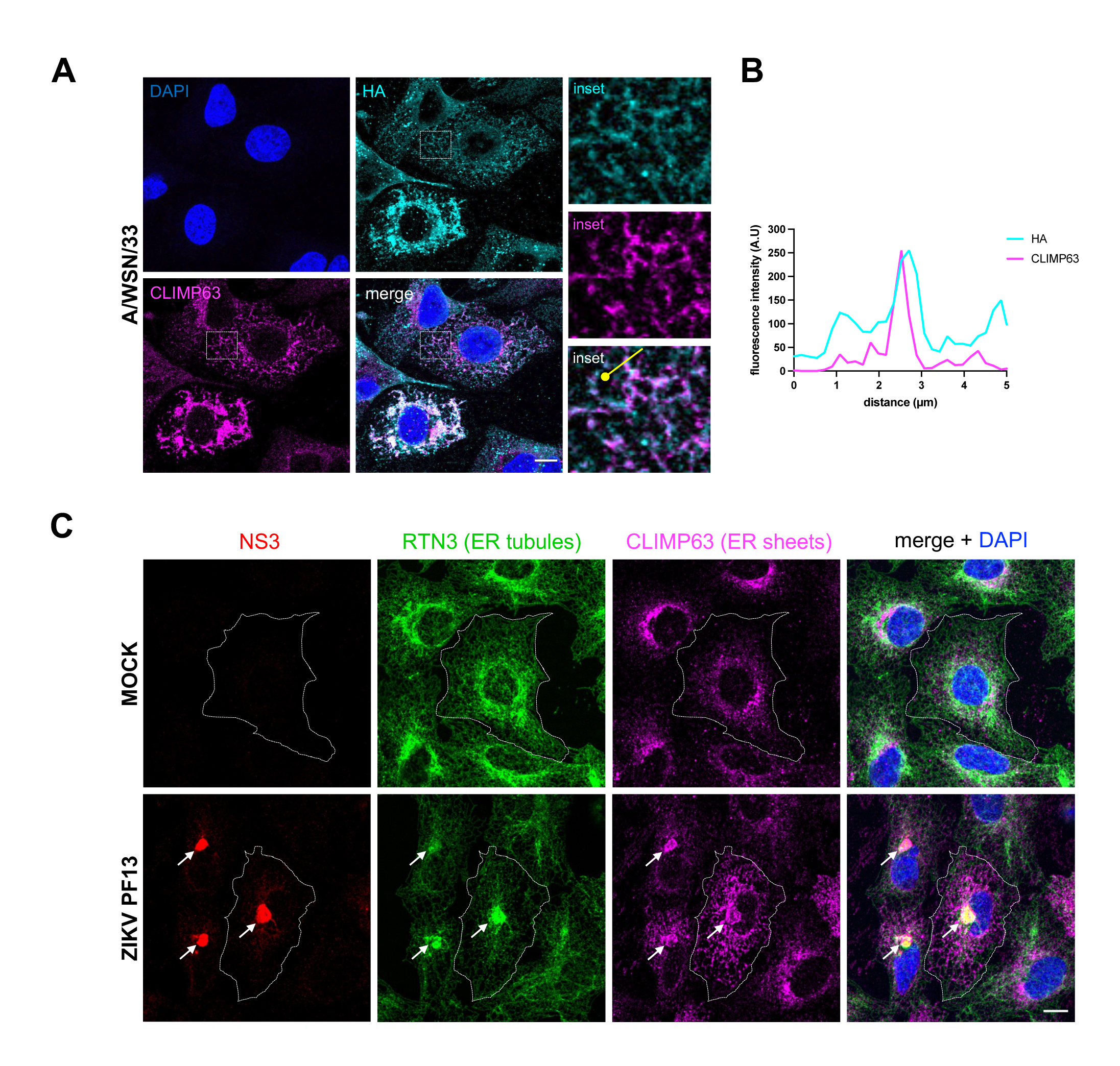

Supplement: S1 Fig — A. A549 cells were infected with WSN at a MOI of 5 PFU/cell for 8 h. Fixed cells were stained for the viral HA and cellular CLIMP63 proteins. Nuclei were stained with DAPI (blue), and cells were imaged with a confocal microscope. Scale bar: 10 µm. B. Fluorescence intensity profile for HA (cyan) and CLIMP63 (magenta) along the yellow line drawn in panel (A) (merge inset), starting from the knob. C. A549 cells were infected with ZIKV PF13 at a MOI of 5 PFU/cell for 24 h, or mock-infected. Fixed cells were stained for the viral NS3 and the cellular RTN3 and CLIMP63 proteins. The RTN3 staining was used to delineate the cell edges. Nuclei were stained with DAPI (blue), and cells were imaged with a confocal microscope. White arrows indicate viral factories surrounded with remodeled ER membranes. Scale bar: 10 µm. The data underlying this figure can be found at https://zenodo.org/records/15682874 (raw images) and S6 File (graphs raw data). (TIF) [file pbio.3002958.s001.tif]

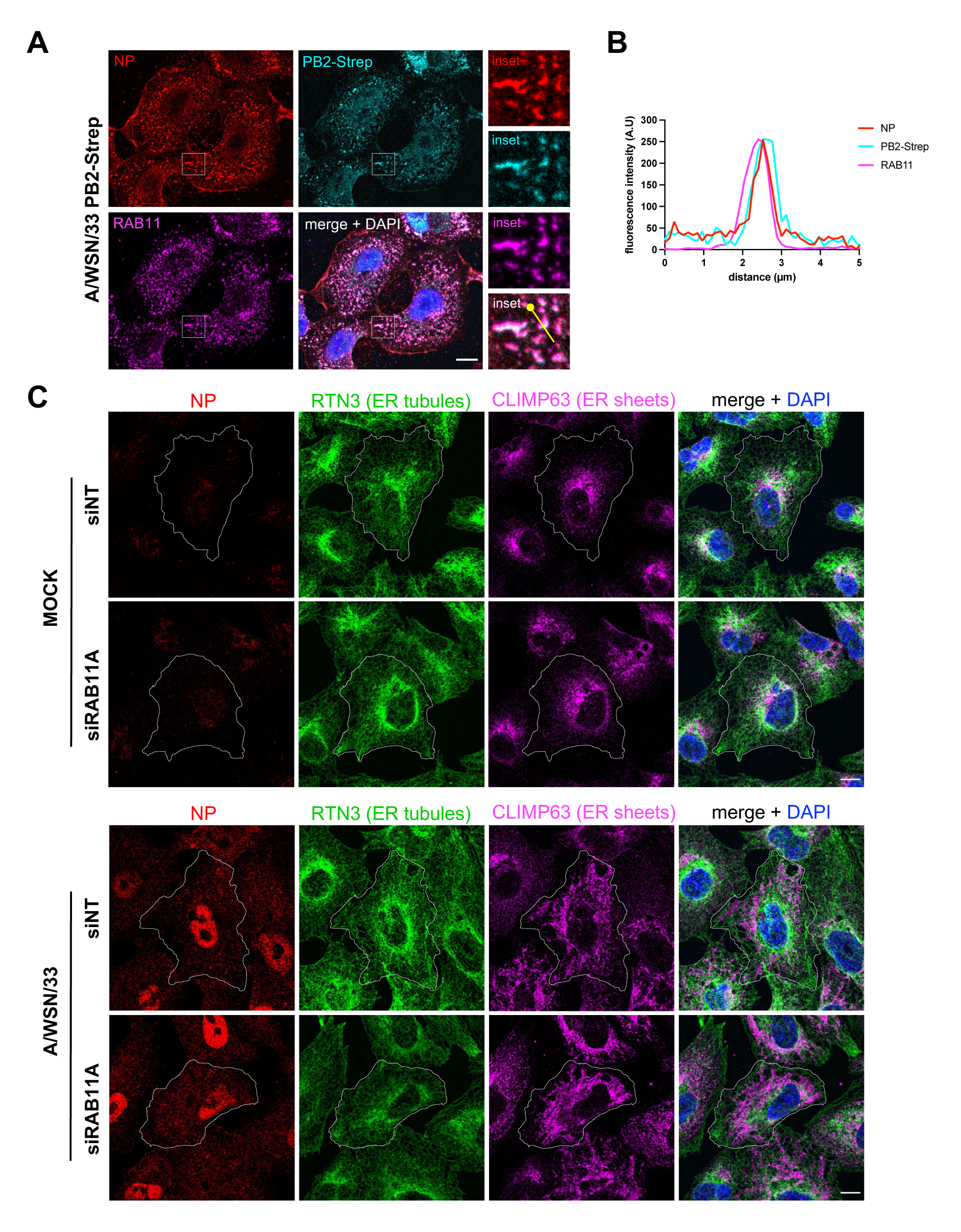

Supplement: S2 Fig — A. A549 cells were infected with the WSN-PB2-Strep virus at a MOI 5 PFU/cell for 8 h. Fixed cells were stained for NP, PB2 (StrepTactin-488) and RAB11. Nuclei were stained with DAPI (blue), and cells were imaged with a confocal microscope. Scale bar: 10 µm. B. Fluorescence intensity profile for NP (red), PB2-Strep-tag (cyan) and RAB11 (magenta) along the yellow line drawn in panel (A) (merge inset), starting from the knob. C. A549 cells were treated with RAB11A-specific or control Non-Target (NT) siRNAs for 48 h, and subsequently infected with WSN at a MOI of 5 PFU/cell for 8 h, or mock infected. Fixed cells were stained for the viral NP and cellular RTN3 and CLIMP63 proteins. The difference in permeabilisation protocols (saponin versus Triton) most likely accounts for the difference in NP signal patterns in this experiment compared to the experiment shown in Fig 2A. Scale bar: 10 µm. The data underlying this figure can be found at https://zenodo.org/records/15682874 (raw images) and S6 File (graphs raw data). (TIF) [file pbio.3002958.s002.tif]

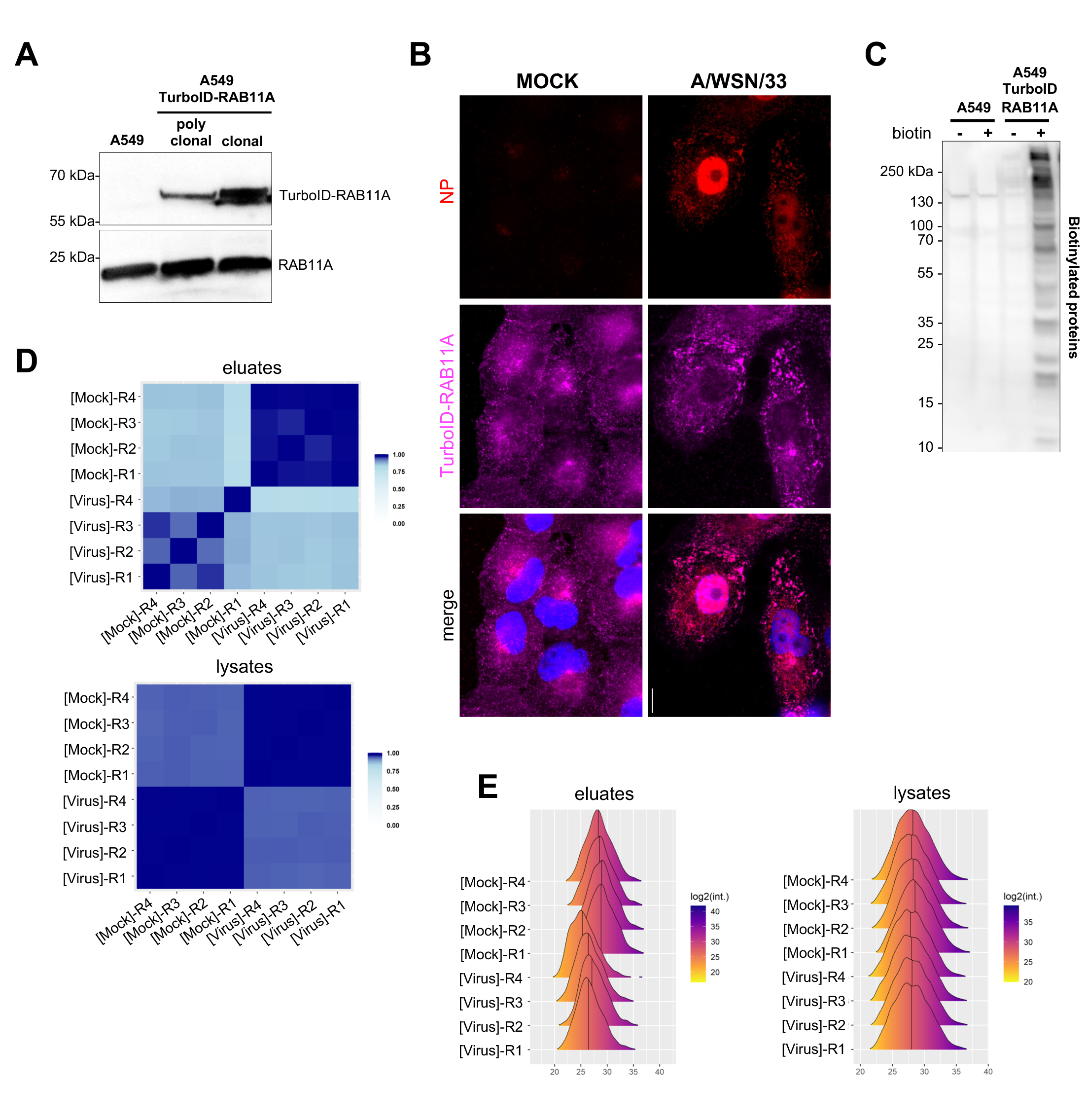

Supplement: S3 Fig — A–C. Characterization of the clonal population of A549 cells stably expressing TurboID-RAB11A. (A) Total cell lysates of A549 parental cells (untransduced), a polyclonal population of transduced A549- TurboID-RAB11A cells, and the clonal population isolated thererof, were analyzed by western blot using an antibody specific for RAB11. The two panels derive from one and the same membrane that was hybridized with an antibody specific for RAB11, thereby allowing to visualize both the endogenous RAB11 and overexpressed TurboID-RAB11A proteins. (B) The clonal A549-TurboID-RAB11A cells were infected with the WSN virus at a MOI of 5 FPU/cell for 8 h. Fixed cells were stained for the HA-tag (3xHA-TurboID-RAB11A, magenta) and the viral NP (red). Nuclei were stained with DAPI (blue), and cells were imaged with an epifluorescence microscope. Scale bar: 10 µm. (C) Total cell lysates of parental A549 cells or clonal A549-TurboID-RAB11A cells, incubated or not for 10 mn at 37 °C in the presence of 50 µM biotin, were analyzed by western blot using streptavidin conjugated with HRP to detect biotinylated proteins. D. Correlation matrices between replicates of eluates (left) and total lysates (right). A correlation matrix represents the Pearson correlation coefficients between each pair of samples computed using all complete pairs of intensity values measured in these samples. Intensity values correspond to TMT-MS2 quantitative relative abundance metrics in the columns titled “Reporter intensity corrected” of the “proteinGroups.txt” file of MaxQuant. The samples identification numbers are indicated in the format “Mock.number of the technical replicate” and “IAV.number of the technical replicate”. Pearson correlation coefficients are indicated in the lower triangular parts of the matrices. In the upper triangular parts, thediameters and gradient colors of the circles are function of these coefficients. E. Distributions of the log2(intensities) for the proteins without missing values in [file pbio.3002958.s003.tif]

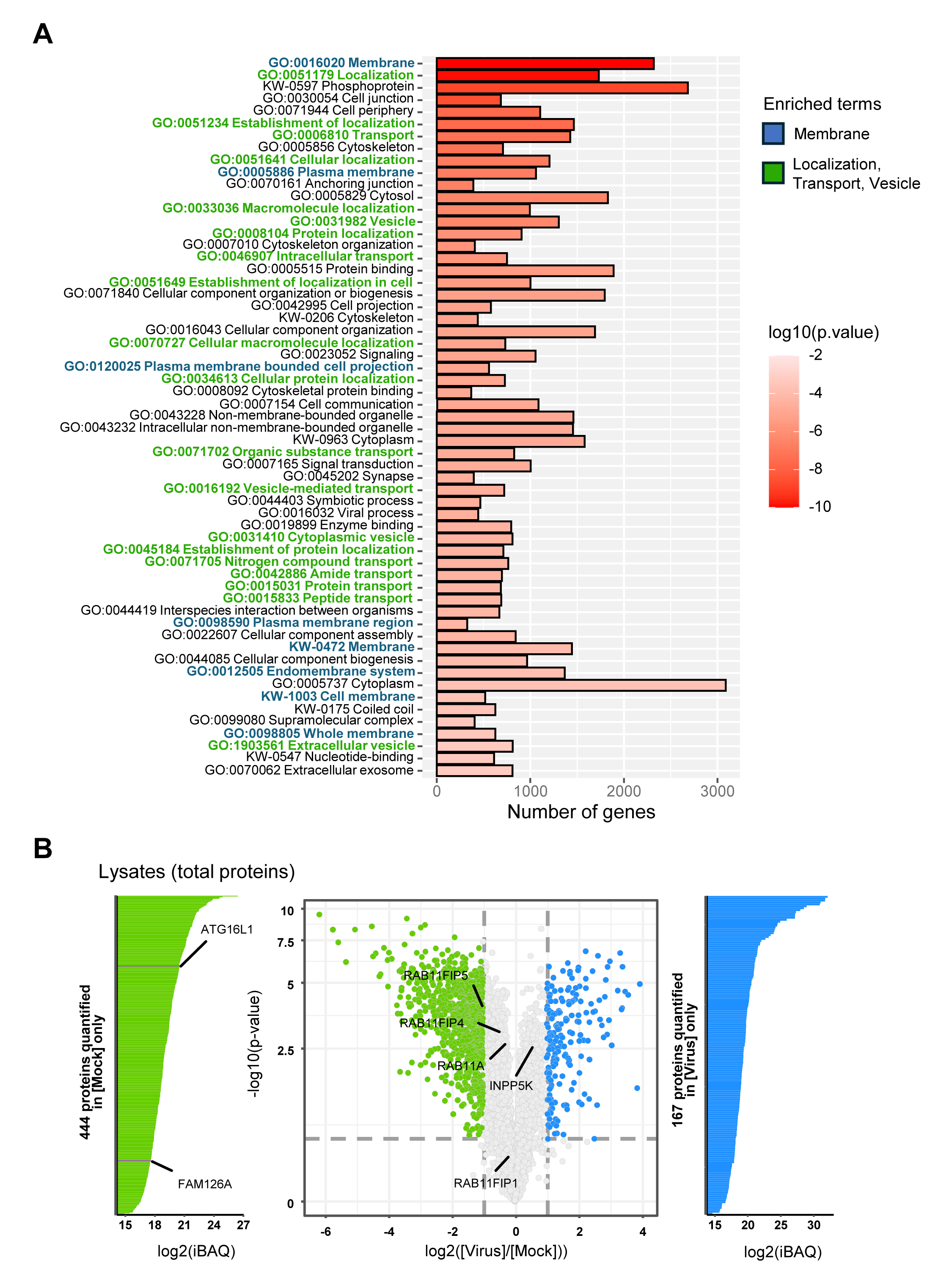

Supplement: S4 Fig — A. Gene Ontology (GO) term enrichment analysis of the set of 3,775 biotinylated proteins identified as being enriched in the biotynylated samples across all conditions and replicates, over a total of 6011 proteins identified in biotinylated eluates and/or total lysates across all conditions and replicates (both sets of proteins are listed in the S1 File). The graph represents the number of genes corresponding to each indicated category (x axis) and the enrichment p-value (color scale). GO terms related to membranes and to intracellular transport/localization/vesicles have been highlighted using blue and green color fonts, respectively. B. Volcano plot showing the log2 fold change (x axis) and its significance (−log10(p-value), y axis) associated to a False Discovery Rate <1%) for each protein (dots) in total lysates from the RAB11A proximity labeling experiment. The log2 fold change refers to the enrichment in WSN-infected (n = 4) versus mock-infected (n = 4) samples. Blue and green dots represent proteins enriched in WSN-infected versus mock-infected samples, and proteins enriched in mock-infected versus WSN-infected samples, respectively. The iBAQ plots shown on the sides of the volcano plot provide additional information on proteins for which no statistical comparison of the abundance could be performed (hence they are not represented in the volcano plot), because they are present only in WSN-infected samples (blue) or only in mock-infected samples (green). The data underlying this figure can be found in S1 File (GO term enrichment) and S3 File (volcano plot). (TIF) [file pbio.3002958.s004.tif]

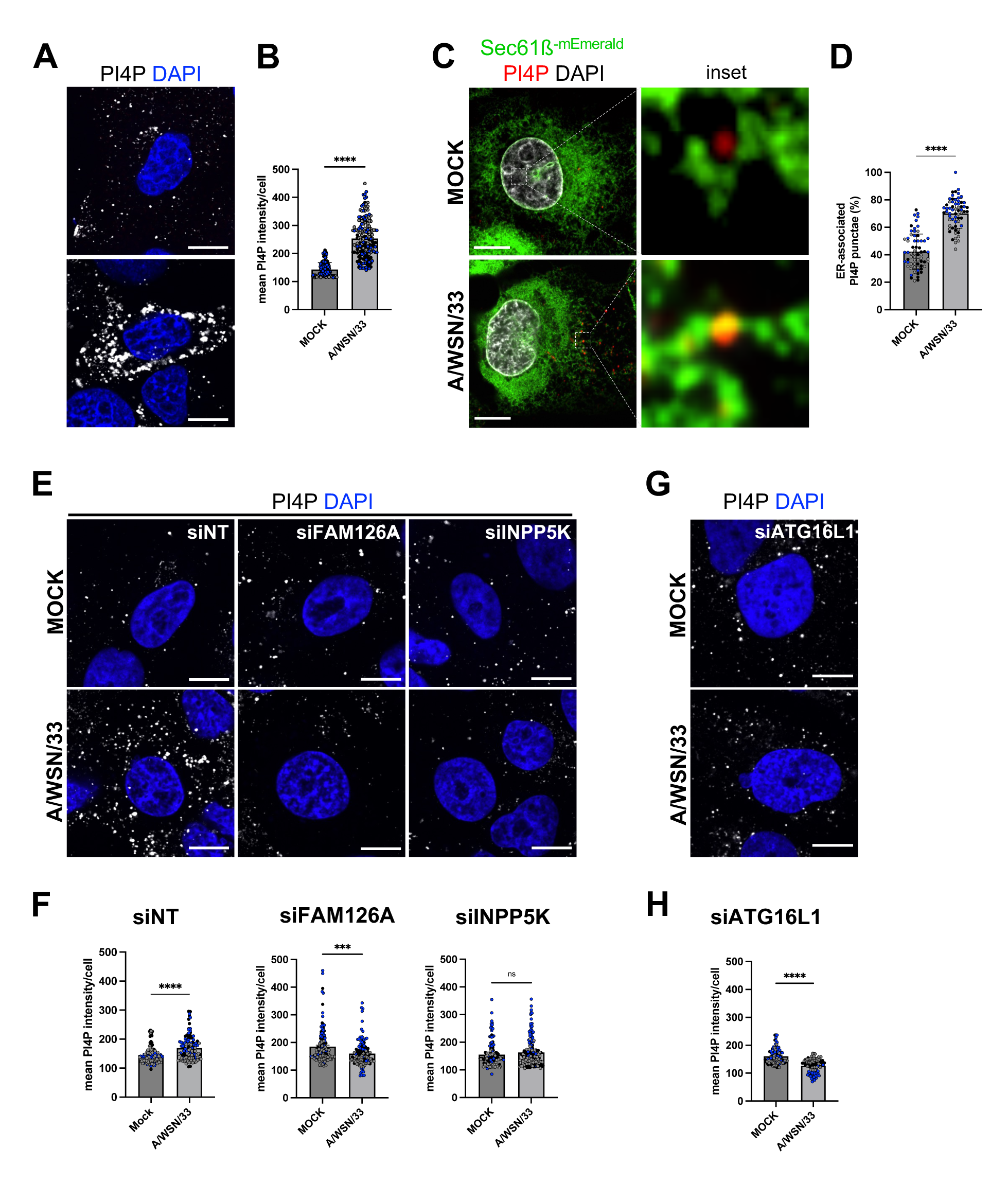

Supplement: S5 Fig — A. A549 cells were infected with WSN at a MOI of 5 PFU/cell for 8 h, or mock-infected. Fixed cells were stained for PI4P with a specific antibody. Nuclei were stained with DAPI (blue), and cells were imaged with a confocal microscope. Scale bar: 10 µm. B. A549 cells treated as in (A) were analyzed with the Fiji software to determine PI4P mean intensity per cell. Each dot represents one cell, and the data from three independent experiments are shown (black, gray and blue dots). The median and standard deviation values are represented (154–170 cells per condition). ****: p-value < 0.0001, unpaired t test. C. U2OS-Sec61ß-mEmerald cells were infected with WSN at a MOI of 5 PFU/cell for 8 h, or mock-infected. Fixed cells were stained for PI4P and nuclei were stained with DAPI (white). Cells were imaged with a confocal microscope. Scale bar: 5 µm. D. U2OS-Sec61ß-mEmerald cells treated as in (C) were analyzed with the Fiji software to determine the percentage of the total PI4P punctae associated to ER in individual cells. Each dot represents one cell, and the data from three independent experiments are shown (black, gray and blue dots). The mean and standard deviation values are represented as histograms (63–66 cells per condition). ****: p-value < 0.0001, unpaired t test. E and G. A549 cells were treated with control non-target (NT) siRNAs or with siRNAs targeting FAM126A, INPP5K (E) or ATG16L1 (G) for 48 h, and subsequently infected with WSN at a MOI of 5 PFU/cell for 8 h, or mock-infected. Fixed cells were stained for PI4P with a specific antibody. Nuclei were stained with DAPI (blue), and cells were imaged with a confocal microscope. Scale bar: 10 µm. F and H. A549 cells treated as in E and G, respectively, were analyzed with the Fiji software to determine the mean intensity of the PI4P signal per cell. Each dot represents one cell, and the data from three independent experiments are shown (black, gray and blue dots). The median and standard deviation values are repres [file pbio.3002958.s005.tif]

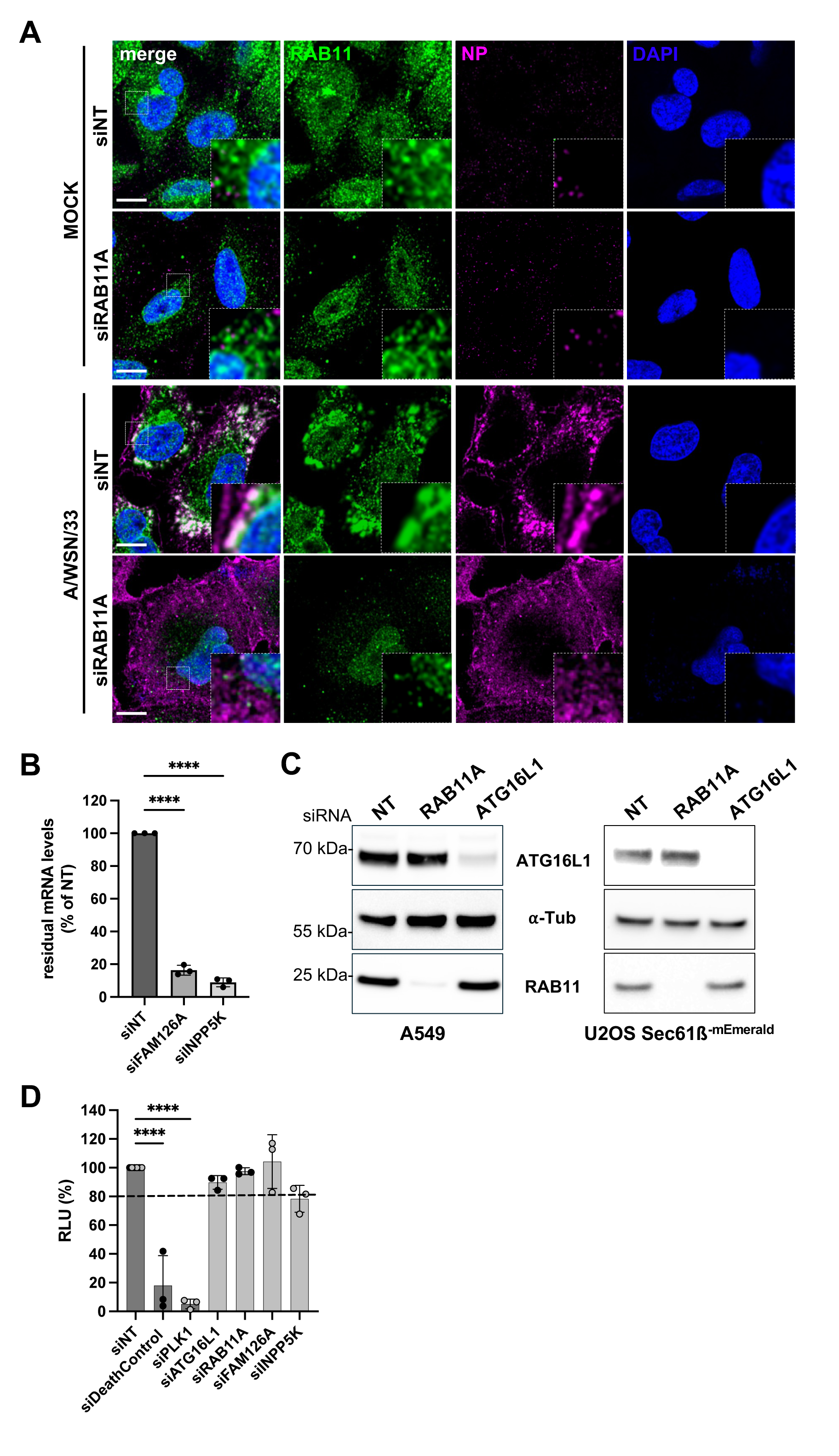

Supplement: S6 Fig — A. A549 cells were treated with RAB11A-specific or control Non-Target (NT) siRNAs for 48 h, and subsequently infected with WSN at a MOI of 5 PFU/cell for 8 h or mock-infected. Fixed cells were stained for the viral NP and the cellular RAB11 proteins. Nuclei were stained with DAPI (blue), and cells were imaged with a confocal microscope. Scale bar: 10 µm. B, C. Knock-down efficiency of siRNA pools. A549 cells were treated with the indicated siRNAs for 48 hours. (B) Total cell lysates were prepared and analyzed by western blot, using the indicated antibodies. (C) Total RNA were extracted and analyzed by RTqPCR using gene specific primers. The residual mRNA levels are expressed as percentages (100%: NT siRNA). Data shown are the mean ± SD of three experiments performed in triplicates. ****: p-value < 0.0001 (one-way ANOVA and Dunnett’s multiple comparisons test, reference: NT siRNA). D. Cell viability upon treatment with siRNA pools. A549 cells were treated with the indicated siRNAs for 48 h and cell viability was determined at 48 hpt using the CellTiter-Glo Luminescent Viability Assay (Promega). The data shown (RLU: Relative Light Units) are expressed as percentages (100%: NT siRNA) and are the mean ± SD of three independent experiments performed in triplicates. Black and white dots correspond to two distinct series of experiments, in which the “Death Control siRNA” (Qiagen) and a siRNA directed against PLK1 were used as positive controls, respectively. The dotted line indicates a 20% reduction in luciferase signal. ****: p < 0.0001 (one-way ANOVA and Dunnett’s multiple comparisons test, reference: NT siRNA, no indication means no significant difference). The data underlying this figure can be found at https://zenodo.org/records/15682874 (raw images), S4 File (uncropped western blots) and S6 File (graphs raw data). (TIF) [file pbio.3002958.s006.tif]

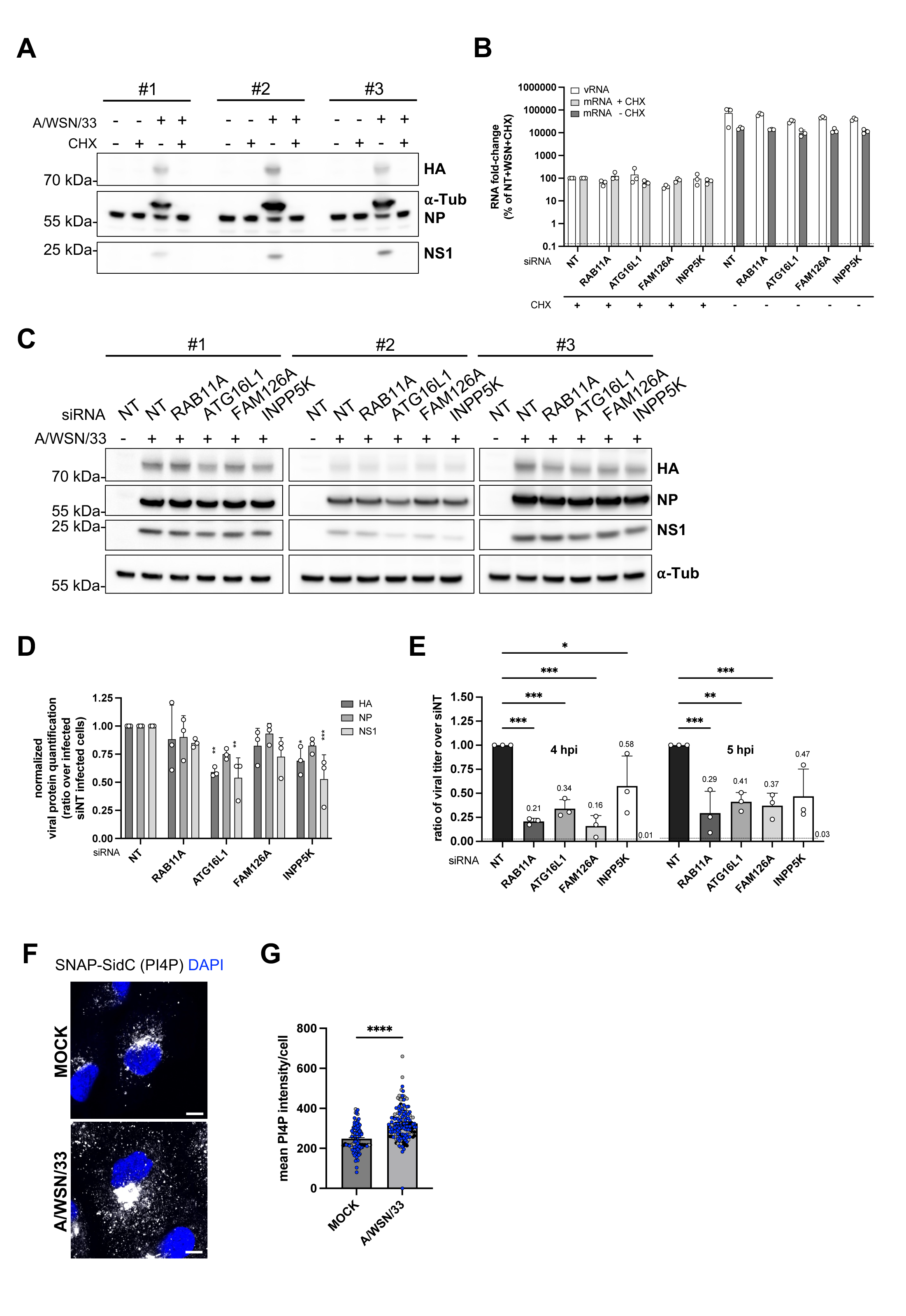

Supplement: S7 Fig — A, B. A549 cells were treated with the indicated siRNA for 48 h and subsequently infected with WSN at a MOI of 5 PFU/cell, in the presence (+) or absence (−) of cycloheximide (CHX). (A) At 6 hpi, total cell lysates were prepared and the steady-state levels of viral proteins were analyzed by western blot, to control for the efficiency of CHX treatment (no viral protein expression). (B) At 6 hpi, total RNA were extracted and the levels of NP-mRNAs and NP-vRNAs were analyzed by strand-specific RTqPCR. The results of three independent experiments, labeled #1, #2, and #3 in (A), are shown. Dotted line: background for mRNA detection in mock-infected cells. Two-way ANOVA with Dunnett’s multiple comparison test; using the siNT without/with CHX samples as a reference for the other without/with CHX samples, respectively, revealed no statistically significant differences. C, D. A549 cells were treated with the indicated siRNA for 48 h and subsequently infected with WSN at a MOI of 5 PFU/cell or mock-infected. At 5 hpi, total cell lysates were prepared and the steady-state levels of viral proteins were analyzed by western blot. (C) Cropped blots of three independent experiments, labeled #1, #2, and #3, are shown. (D) The signals for HA, NP ad NS1 were normalized over the α-tubulin signal (α-Tub) and expressed as percentages (100%: NT siRNA). The data shown are the mean ± SD of the three independent experiments. *: p-value < 0.05, **: p-value < 0.01, ***: p-value < 0.001 (two-way ANOVA with Dunnett’s multiple comparison test, reference: siNT). E. A549 cells were treated with the indicated siRNA for 48 h and subsequently infected with WSN at a MOI of 5 PFU/mL. At 3 or 4 hpi, the culture medium was extensively washed away four times to remove the viral input and replaced with fresh medium. One hour later, i.e., at 4 or 5 hpi, respectively, the supernatants were collected. The last wash (residual input) and collected supernatants were titrated by a plaque assay. The titers were nor [file pbio.3002958.s007.tif]

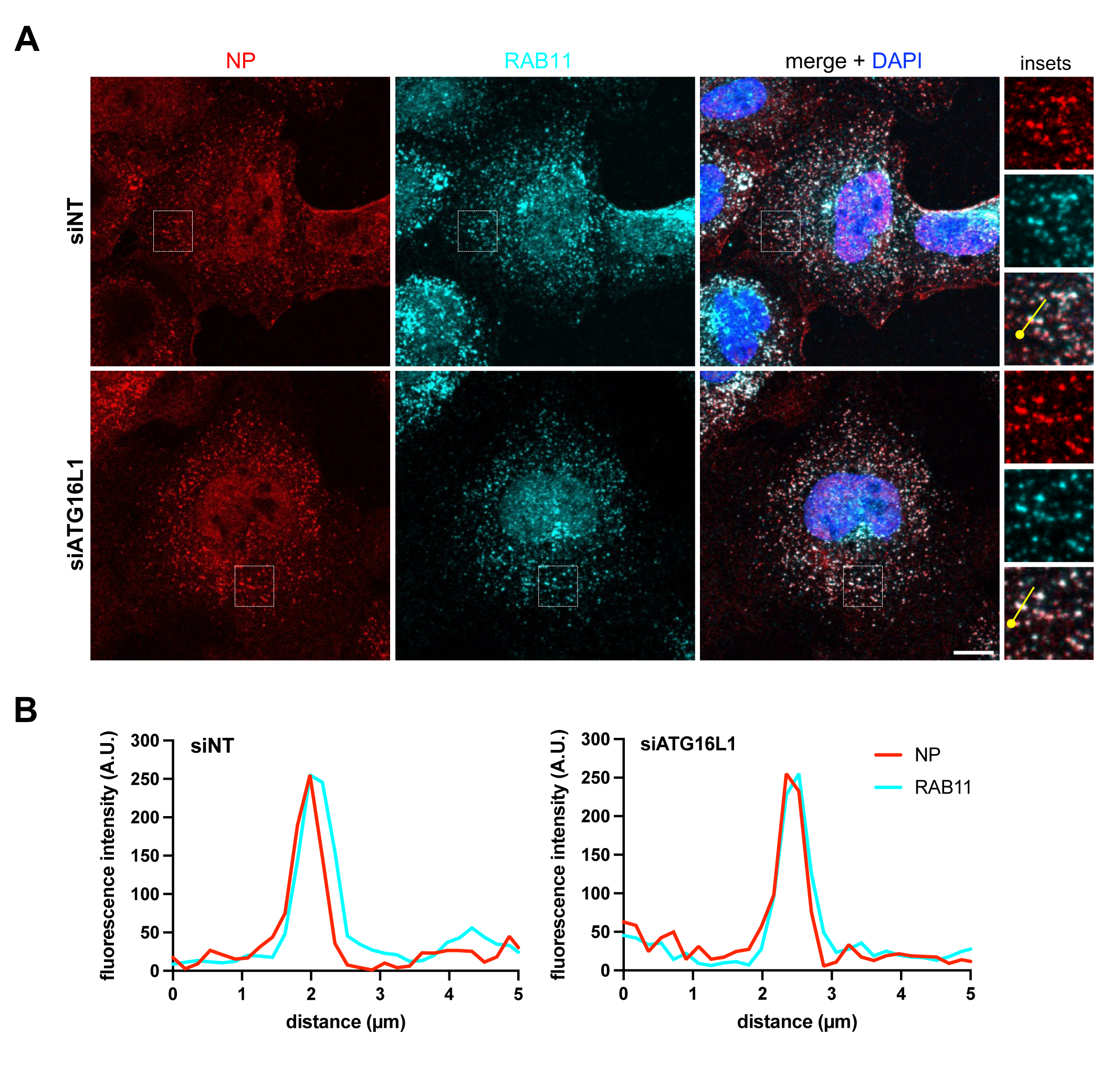

Supplement: S8 Fig — A. A549 cells were treated with control NT or ATG16L1 siRNA for 48 h and subsequently infected with WSN at a MOI of 5 PFU/cell for 8 h. Fixed cells were stained for viral NP and cellular RAB11 proteins. Nuclei were stained with DAPI (blue), and cells were imaged with a confocal microscope. Scale bar: 10 µm. B. Fluorescence intensity profile for NP (red) and RAB11 (cyan) along the white line drawn in panel (A) (merge inset), starting from the knob. The data underlying this figure can be found at https://zenodo.org/records/15682874 (raw images) and S6 File (graphs raw data). (TIF) [file pbio.3002958.s008.tif]

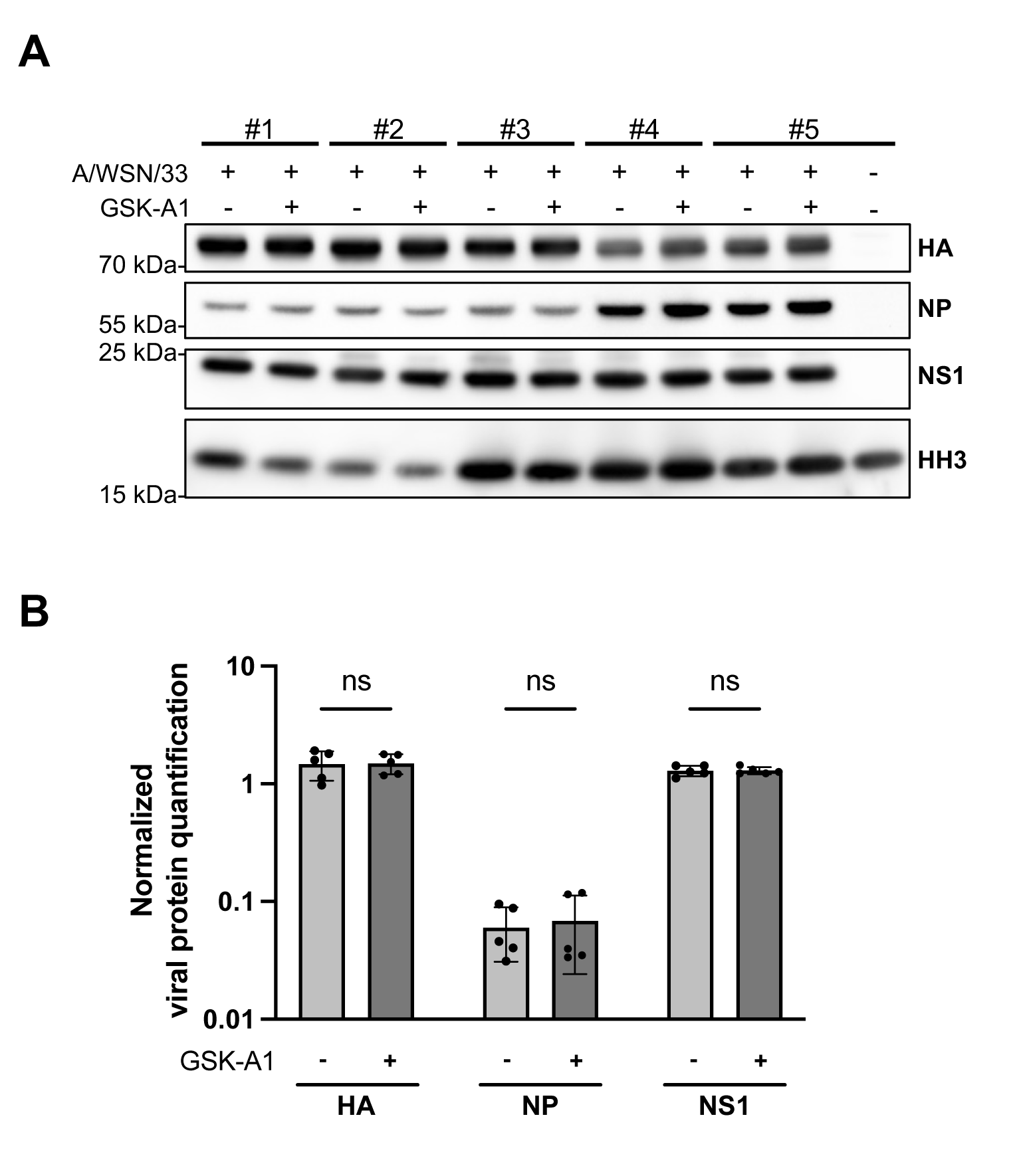

Supplement: S9 Fig — A. A549 cells were infected with WSN at a MOI of 5 PFU/cell. Two hours later, the GSK-A1 drug was added at a final concentration of 100 nM. At 6 hpi, total cell lysates from five independent experiments (#1 to #5) were prepared and analyzed by western blot, using the indicated antibodies. Cropped blots are shown. B. The signals for the viral HA, NP and NS1 proteins and the RTN3 protein were normalized over the histone H3 (HH3) signal and expressed as percentages (100%: mock-infected cells). The data shown are the mean ± SD of three independent experiments (two-way ANOVA with Dunnett’s multiple comparison test, reference: mock-infected cells, no indication means no significant difference). The data underlying this figure can be found in S4 File (uncropped western blots) and S6 File (graphs raw data). (TIF) [file pbio.3002958.s009.tif]
