# Supplementary material for: Influenza A virus induces PI4P production at the endoplasmic reticulum in an ATG16L1-dependent manner to promote the egress of viral ribonucleoproteins
Source: PLoS Biol. 2025 Jul 16;23(7):e3002958. doi: 10.1371/journal.pbio.3002958 (PMC12286409; doi:10.1371/journal.pbio.3002958)
Supplement: Attachment — Submitted filename: Point-by-point response to reviewers_20250528.pdf [file pbio.3002958.s016.pdf]

**Response to reviewers of manuscript PBIOLOGY-D-24-03404**

**Influenza A virus-induced production of PI4P at the endoplasmic reticulum involves ATG16L1 and promotes the egress of viral ribonucleoproteins**

We would like to thank the three reviewers for acknowledging the significance and novelty of our study and for their fair and constructive comments. In the revised version of the manuscript, additional data have been included and the text has been modified in order to address their concerns and suggestions.

A revised version of the manuscript with highlighted changes is provided.

Below is the response to a major point raised by both reviewers #1 and #3, followed by a point-by-point response to each of the reviewers comments.

**MAJOR POINT RAISED BY REVIEWERS #1 AND #3.**

Reviewers #1 and #3 expressed concerns that "*some of the effects on reduced viral progeny release resulting from the absence of ATG16L1 or PI4P associated enzymes could be due to effects on viral entry or degradation*" (Reviewer #1) and that we should "*perform synchronized infections at a high MOI, harvest virus after a single cycle of infection, and show that protein production is unaffected by western blot, to rule out a defect in any of the stages prior to assembly/trafficking*" (Reviewer #3).

To adress these concerns, we performed infections at a high MOI (5 PFU/cell) in cells transfected with siRNAs targeting RAB11A, ATG16L1, FAM126A or INPP5K, and we examined the progression of the viral cycle using three types of read-out:

Read-out #1) the expression of viral transcripts at 6 hpi in the presence of cycloheximide, as measured by strand-specific RTqPCR: in these conditions, no translation of viral proteins and therefore no replication of vRNPs can occur. The detectable viral transcripts result from the parental vRNPs serving as template for primary transcription. There we monitor the efficiency of early stages of the viral life cycle, i.e. viral uptake, nuclear import of vRNPs and primary transcription.

We found that the earliest stages of the viral life are not significantly affected by any of the above mentioned knock-downs, as indicated by unchanged levels of accumulation of primary NP-mRNAs in the presence of cycloheximide (**Figure S7A-B** in the revised version of the manuscript).

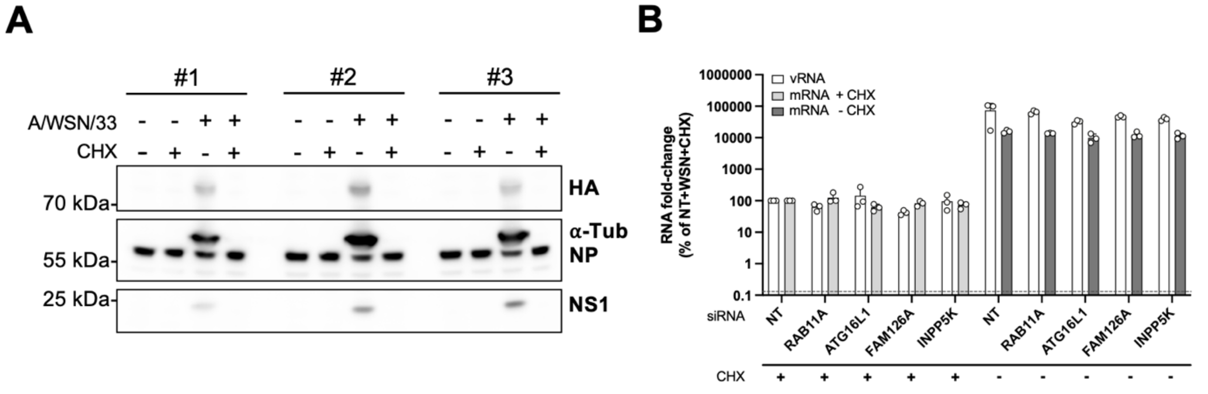


**Figure S7A-B (revised manuscript)**: A549 cells were transfected with the indicated siRNA for 48h and subsequently infected with WSN at a MOI of 5 PFU/cell, in the presence (+) or absence (-) of cycloheximide (CHX). (A) At 6 hpi, total cell lysates were prepared and the steady-state levels of viral proteins was analyzed by western-blot, to control for the efficiency of CHX treatment (no viral protein expression). (B) At 6 hpi, total RNA were extracted and the levels of NP-mRNAs and NP-vRNAs were analyzed by strand-specific RTqPCR. The results of three independent experiments, labelled #1, #2 and #3 in (A), are shown. Dotted line : background for mRNA detection in mock-infected cells. Two-way ANOVA with Dunnet's multiple comparison test ; using the siNT without/with CHX samples as a reference for the other without/with CHX samples, respectively, revealed no statistically significant difference.

Read-out #2) the expression of viral transcripts and proteins at 5-6 hpi in the absence of cycloheximide, as measured by strand-specific RTqPCR and western-blot, respectively. There we monitored the viral cycle up to an intermediate stage.

The levels of NP-mRNAs and genomic negative-sense NP-vRNAs were not significantly affected by any of the tested siRNAs (**Figure S7A-B** in the revised version of the manuscript, shown above). Consistently, no significant changes in steady-state levels was observed for the NP protein; however, a moderate (< 2-fold) but reproducible decrease was observed for the NS1 and HA proteins in ATG16L1-KD and in INPP5K-KD cells (**Figure S7C-D** in the revised version of the manuscript).

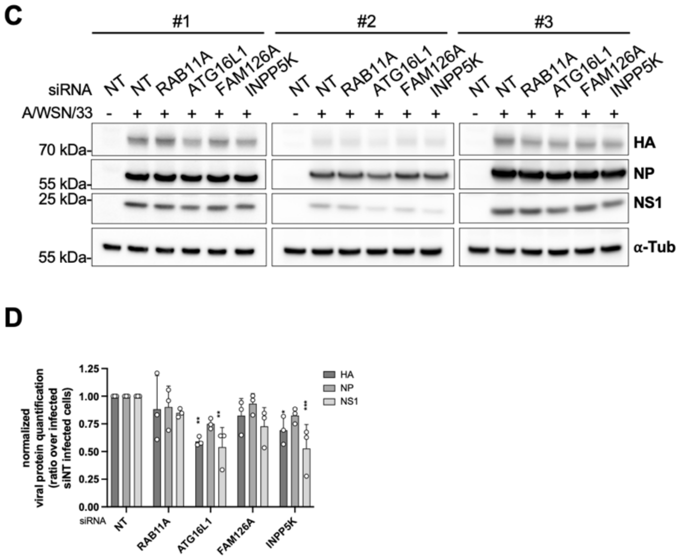


**Figure S7C-D (revised manuscript).** A549 cells were transfected with the indicated siRNA for 48h and subsequently infected with WSN at a MOI of 5 PFU/cell or mock-infected. At 5hpi, total cell lysates were prepared and the steady-state levels of viral proteins was analyzed by western-blot. (C) Cropped blots of three independent experiments, labelled #1, #2 and #3, are shown. (D) The signals for HA, NP ad NS1 were normalized over the $\alpha$-tubulin signal and expressed as percentages (100% : NT siRNA). The data shown are the mean ± SD of the three independent experiments. * p<0.05, ** p<0.01, ***p<0.001 (two-way ANOVA with Dunnet's multiple comparison test, reference : siNT).

Read-out #3) the production of infectious viral particles, as measured by a plaque assay. There we monitored the viral cycle up to the latest stages. At 8 hpi, we did not observe any significant impact of any of the knock downs, not even the knock-down of RAB11A, whose role in vRNPs transport is undisputable. We hypothesized that at a high MOI, when the major vRNP transport route is altered, enough vRNPs are transported using less efficient routes (for example, using RAB11B instead of RAB11A) so that a plateau in infectious virus production is reached at 8 hpi. Therefore we measured the egress of infectious viral particles at earlier time points and observed a reproducible decrease in the production of viral particles (**Figure S7E** of the revised version of the manuscript).

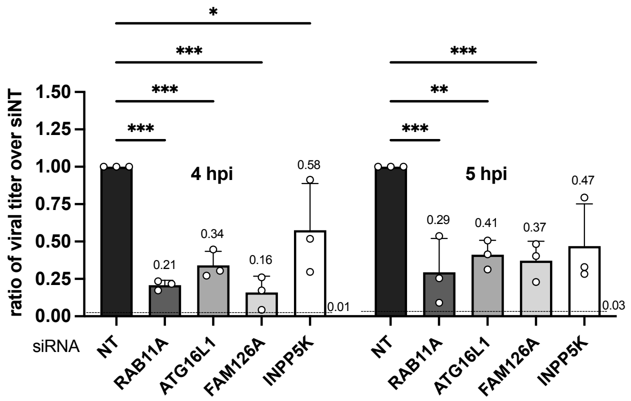


**Figure S7E (revised manuscript).** A549 cells were transfected with the indicated siRNA for 48h and subsequently infected with WSN at a MOI of 5 PFU/mL. The culture medium was washed away and replaced with fresh medium at 3 or 4 hpi. At 4 or 5 hpi, respectively, the supernatants were collected and the infectious titers were determined by plaque assay. The titers were normalized over the control (NT siRNA). The data shown are the mean ± SD of three independent experiments. * p<0.05, ** p<0.01, ** p<0.001 (two-way ANOVA with Dunnet's multiple comparison test, reference : siNT). The dotted lines represents the average ratio value for residual inputs.

Importantly, a virus-mediated increase in total levels of PI4P is already observed as early as 4 hpi (**Figure 7F-G** of the revised manuscript).

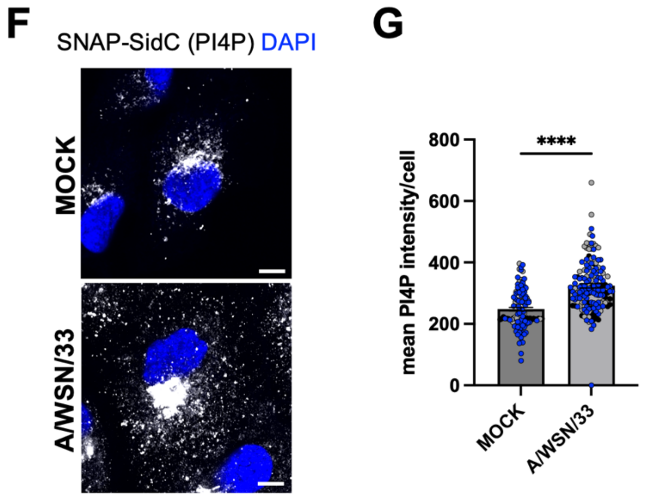


**Figure S7F-G (revised manuscript).** A549 cells were infected with WSN at a MOI of 5 PFU/mL for 4 h, or mock-infected. (F) Fixed cells were stained for PI4P using the SNAP-SidC probe (white). Nuclei were stained with DAPI (blue) and cells were imaged with a confocal microscope. Scale bar : 10µm. (G) A549 cells treated as in (F) were analyzed using the Fiji software to determine the mean intensity of the PI4P signal per cell. Each dot represents one cell, and the data from three independent experiments are shown (black, grey and blue dots). The median and standard deviation values are represented (39-155 cells per condition).The data shown are the mean ± SD of the three independent experiments. **** p<0.0001, unpaired t-test.

Overall, these new data demonstrate that the absence of RAB11A, ATG16L1 or PI4P-associated enzymes induces no significant defect at the early stages of the viral cycle, and reduces the production of infectious viral particles. In the absence of RAB11A and FAM126, the steady-state accumulation levels of the viral proteins are unchanged, clearly pointing to a defect at the latest stages (vRNP transport/assembly) of the viral cycle. We cannot formally rule out that the slight reduction in viral proteins steady-state levels observed in the absence of ATG16L1 and INPP5K could partially account for the reduction in viral progeny. This possiblity does not call into question the other data provided in our manuscript, which demonstrate that the absence of ATG16L1 results in a defect in vRNP transport and exit (Figure 5 and 6 of the initial and the revised version).

These additional data are described page 12 lines 30-42 and page 13 lines 1-4 of the revised version of the manuscript, and are discussed page 13 lines 7-14.

**REVIEWER #1**

This manuscript is well written and contains high quality data. It includes a number of correlative data showing that influenza virus infection leads to changes in phosphoinositide metabolism and recruitment to ER domains. The effects on PI metabolism seem to require the activities of PI4P processing enzymes as well as RAB11A and ATG16L1. Many of the observations made by the authors are interesting, however their functional relevance or underlying mechanisms have not been fully explored. I mainly suggest rewording and revisiting some of the conclusions drawn by the authors.

As explained in more details below, we performed additional experiments to better support our claims and we rephrased some of our conclusions.

RAB11A KD, but not ATG16L1 KD, is shown to abolish the punctate staining of viral NP (Fig.'s 2A and 5A). This observation has not been fully explored. Perhaps the authors could at least comment on this finding which seems to stand out in their data.

We thank the reviewer for drawing our attention to this point. We agree that the differences observed between RAB11A KD and ATG16L1 KD regarding the viral NP cytosolic staining (a proxy for vRNP staining) at late time points in infection raise an important question about the sequential and/or synergistic roles of RAB11A and ATG16L1 in vRNP transport.

The striking alteration of NP staining upon RAB11A, i.e. a shift from a punctate to a diffuse distribution, has been reported in several publications (Amorim et al., Journal of virology 2011 ; Eisfeld et al., Journal of Virology 2011, Momose et al., PLoS ONE 2011). There is evidence that the NP- and RAB11A positive punctae correspond to clusters of vRNP transport vesicles (Vale-Costa et al, Journal of Cell Science 2016).

As RAB11A-positive membranes at the vicinity of the ER serve as a platform for the formation of vRNP-coated vesicles (De Castro Martin et al., Nature communications 2017 ; Alenquer et al., Nature communications 2019), a very likely explanation for the fact that RAB11A KD abolishes the NP punctate staining is that vRNP-coated vesicles fail to be produced, hence vRNPs remain dispersed in the cytoplasm, particularly along ER membranes (Eisfeld et al., Journal of Virology 2011, and the present manuscript, Figure 2A and Figure 5A).

In ATG16L1 KD cells, RAB11A-positive membranes remain present. The fact that cytosolic NP staining remains punctate (Figure 5A of our manuscript) indicates that in the absence of ATG16L1, RAB11A-positive membranes can still support ICV biogenesis. However, as shown in Figure 6, vRNP transport to the plasma membrane is strongly altered in ATG16L1 KD cells. We thus hypothesize that in the absence of ATG16L1 and a proper PI4P signaling, which is known to be involved in membrane identification and membrane vesicular trafficking, the vRNP-coated vesicles formed in ATG16L1 KD cells are not efficiently transported to the plasma membrane.

Overall, our observations confirm that RAB11A is critical for the biogenesis of vRNP-coated vesicles, and indicate that in addition to RAB11, ATG16L1 and the PI4P signaling machinery are required for the vRNP-coated vesicles to be fully functional and efficiently transported towards the plasma membrane.

These comments have been included in the revised version of the manuscript (page 14 lines 34-43). The introduction (page 5 lines 14-16) and the legend of Figure 7 (working model, page 20 lines 8-13) were revised accordingly.

Figures 4&6: Could some of the effects on reduced viral progeny release resulting from the absence of ATG16L1 or PI4P associated enzymes be due to effects on viral entry or degradation? Similarly, would it be possible that, in the presence of siRNA targeting FAM126A, NPP5K and ATG16L1, the suppressed increase in PI4P levels is due to changes in viral uptake? These possibilities have not been explored or commented on in this manuscript.

This important point, also raised by Reviewer #3, has been adressed in details above (please see pages 1-3 of the present rebuttal).

Additional comments:

The authors say that "comparable steady-state levels for the recombinant TurboID-RAB11A and the endogenous RAB11A proteins, as assessed by western-blot (S3A Fig)." not clear this statement can be derived from the shown blots unless the authors can present an uncropped blot containing both endo-RAB11A and exogenous protein.

In the S3A Fig legend of the revised version of the manuscript, we made it clear that the two panels derive from one and the same membrane that was hybridized with an antibody specific for RAB11, thereby allowing to visualize both the endogenous RAB11 and overexpressed TurboID-RAB11A proteins.

Uncropped blots were provided with the initial submission of the manuscript, and are provided with the revised version as well. The statement “Uncropped immunoblots are shown in S4 File” has been added in the Materials and Methods section (page 28, line 30).

The conclusion "this proviral activity of ATG16L1 is most likely mediated by the control of local PI4P production on ER membranes." should be toned down as the authors show no direct evidence for this and the conclusion is based on correlative data.

We agree with the reviewer’s comment that, although we show an increase of both ATG16L1 and PI4P at the ER membranes, we do not demonstrate that the proviral activity of ATG16L1 is mediated by the control of local PI4P on ER membranes. Therefore we have toned down this conclusion, (page 15 lines 11-14 of the revised version of the manuscript), as follows :

*« Altogether, our observations indicate that ATG16L1 is involved at late stages of IAV life cycle and promotes vRNP transport to the plasma membrane, concomitant with or possibly through the control of local PI4P production on ER membranes »*

Fig 6C; there seems to be an increase of NP-associated puncta; is this due to reduce viral exit/degradation?

We agree with the reviewer's observation, and in the revised version of the manuscript we provide a quantification of this increase in NP-staining at 8 hpi upon ATG16L1 depletion. The data are included as an additional **Figure 6C** in the revised version of the manuscript, and described page 15 lines 3-9).

Interestingly, a similar increase in NP staining was recently reported upon depletion of ATG9A, also a member of the ATG family of proteins, and also found to contribute to the intracellular transport of influenza vRNPs (Vale-Costa et al., PLoS Biology 2023).

The most likely hypothesis is that the observed increase in NP staining is linked to reduced vRNP exit and subsequent intracellular accumulation of vRNPs.

**C**

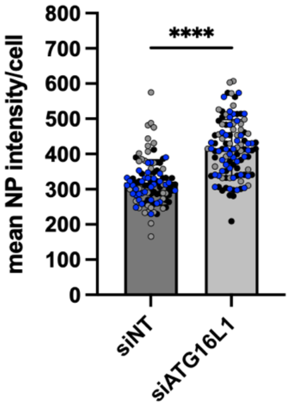


**Figure 6C (revised version).** A549 cells treated as in (A) were analyzed with the Fiji software to determine the mean intensity of NP signal per cell. Each dot represents one cell, and the data from three independent experiments are shown (black, grey and blue dots). The median and standard deviation values are represented (28 to 40 cells per experiment). **** : p-value <0.0001, unpaired t-test.

The conclusion that influenza virus trafficking is perturbed in the absence of ATG16L1 or PI4P processing should be supported by colocalisation experiments with various endocytic compartments. Alternatively, such conclusions can be toned down.

Our study focuses on the ER and RAB11A-positive membranes. We respectfully feel that examining various endocytic compartments upon influenza-virus infection beyond the RAB11-positive compartment represents a whole topic in itself and is beyond the scope of the present study. However, to address the reviewer's comment, we highlight in the revised version of the manuscript the RAB proteins and RAB effector proteins whose abundance in RAB11A interactome is decreased (28 proteins) or increased (3 proteins) upon infection. They are listed in an additional tab of S2 File, and are referred in the discussion (page 17 lines 15-21 of the revised version), as follows :

*« In addition, the abundance of 28 RAB proteins (notably RAB5A, RAB5C, RAB7A) and RAB effector proteins (for instance RABGEF1) was found to be decreased in the RAB11A proximity interactome of infected cells compared to control cells, while the opposite trend was observed for RAB2B, RAB23 and RAB43 (S2 File). Overall, our observations reinforce the notion that in IAV-infected cells, the intracellular localization, interaction with other endocytic and exocytic compartments and function of RAB11A-positive membranes are distinct from those of recycling endosomes. »*

We also further documented how the transport of influenza vRNPs is perturbed in the absence of ATG16L1. We provide new data showing that the ability of vRNPs to co-localize with RAB11 is not affected upon ATG16L1 depletion (**Figure S8** of the revised manuscript). This observation is consistent with the observation (discussed above) that the punctate NP staining, thought to correspond to vRNP-coated vesicles formed at RAB11A-positive membranes, is not altered. It reinforces the notion that ATG16L1 is critical, not for the biogenesis of vRNP-coated vesicles, but for their transport towards the plasma membrane.

To avoid any misinterpretation or overstatement, in the revised version of the manuscript we replaced "trafficking" by "transport to the plasma membrane".

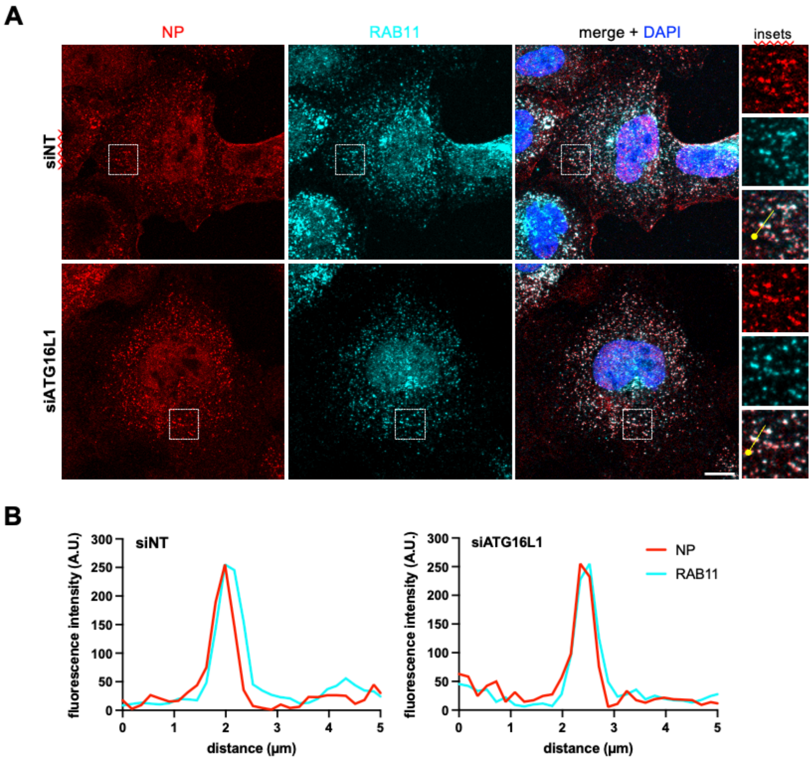


**Figure S8 (revised version).** Impact of ATG16L1 depletion on the distribution of RAB11A and vRNPs (related to figure 5). A. A549 cells were treated with control NT or ATG16L1 siRNA for 48h and subsequently infected with WSN at a MOI of 5 PFU/cell for 8 h. Fixed cells were stained for viral NP (red) and cellular RAB11 (cyan) proteins. Nuclei were stained with DAPI (blue) and cells were imaged with a confocal microscope. Scale bar : 10µm. B. Fluorescence intensity profile for NP (red) and RAB11 (cyan) along the white line drawn in panel A (merge inset), starting from the knob.

The authors conclude on pg.17 that "mode of action of ATG16L1 could be unrelated to canonical autophagy" without giving any experimental evidence. They suggest that the "RAB11A interactome" did not detect any canonical autophagy players, such as ATG5-12 or WIPI2. This is not a valid reasoning as the RAB11A proximity labelling depends on the location of the tag and distance to neighboring proteins and is not necessarily a reflection of the interactome. This conclusion should be revisited.

We agree with the reviewer’s criticism. The discussion regarding canonical versus autophagy-independent has been revised accordingly, page 19 lines 12-29, as follows

*« Although ATG16L1 is a key component of the autophagic machinery, and a cooperation between ATG16L1 and RAB11A-positive membranes has been described in the context of the canonical autophagic response to starvation stress [46], there is growing evidence that ATG16L1 also plays a significant role in membrane-based processes that are not autophagic [47]. In IAV-infected cells, the autophagy process is initiated but its full completion is prevented. The viral NS1 protein was found to repress the formation of autophagosomes in A549 and HeLa cells [48]. In A549 cells constitutively expressing GFP-LC3, autophagosomes were detected but their fusion with lysosomes was inhibited by the viral M2 protein [49], and there is evidence that M2 abrogates the TBC1D5-RAB6 interaction to escape lysosomal degradation [50]. Interestingly, a recent study reports that silencing of canonical autophagic proteins such as ATG2 or ULK1 does not impair influenza vRNP trafficking, whereas silencing of another component of the autophagy machinery, ATG9A, does so [9]. An increase in intracellular NP at late time points was observed upon depletion of ATG9A [9] (similar to what we observed upon depletion of ATG16L1) and ATG9A was found to regulate membrane-microtubule contacts in IAV-infected cells, leading the authors to conclude that this mechanism is unrelated to canonical autophagy. Likewise, we speculate that in the context of an IAV infection, the mode of action of ATG16L1 is most likely unrelated to canonical autophagy. »*

**REVIEWER #2**

The study by Alemany et al. aims to shed light on the still poorly understood process of influenza A virus (IAV) vRNP transport to assembly sites. By performing proximity labelling of RAB11A during infection the authors reveal that IAV can modulate levels of the phosphoinositide PI4P. They show that this modulation of PI4P levels is required for vRNP transport. They also reveal a role for ATG16L1 in regulating PI4P dynamics and coordinating RAB11A-dependent vRNP transport.
The data are presented in a very clear manner and support the conclusions drawn, with one exception (see points on figure 4). In my opinion, this work represents an important step forward in our understanding of the cell biology of IAV infection.

**Major comments:**

- Fig. 4A-B: Immunofluorescence-based detection is useful for analyzing co-localization but in my opinion, it is not ideal for quantification of protein or PI4P levels given its limited dynamic range. Can the authors confirm the increase in PI4P levels by a different method? This would strengthen this crucial finding of the manuscript substantially.

Quantifying the expression level of specific lipids is not trivial, because they are far more labile than proteins and because of the paucity of reagents suitable for quantification.

Fluorescence-based methods, although they show limitations, are considered as the best existing methods for phosphoinositides detection and quantification. Fluorescence-based detection of PI4P is usually achieved by one of these two means

i) by overexpressing a PI4P-binding protein domain : not the best method, as the fine-tuned balance between PI4P lipids and their metabolizing enzymes can be disturbed
ii) by using a PI4P-specific antibody : the method we used in Fig 4A-B of the initial version of the manuscript

We took opportunity of a novel biosensor recently developped by David Murray (Maib et al, J Cell Biol, 2024) based on the SidC factor, a natural binder for PI4P, fused to the self-labeling SNAP-tag. This biosensor allows for a precise detection of PI4P pools including a strong Golgi-associated fraction which is barely detected with the antibody. To adress the reviewer's comment, we repeated all the experiments shown in Figure 4 of the initial version of the manuscript, using the SNAP-SidC biosensor instead of the anti-PI4P antibody. The two datasets are very consistent, as the SNAP-SidC biosensor also reveals a striking increase in PI4P levels in the cytoplasm upon IAV infection. This increase is abrogated in the absence of ATG16L1 or the PI4P related enzymes.

In the revised version on the manuscript, **Figure 4** now shows the data produced with the SNAP-SidC biosensor, while the data produced with the PI4P-specific antibody are shown as **Supplemental Figure S5**. The text has been modified accordingly, page 11 lines 19-29.

- Fig. 4: The images seem to show much larger differences between mock and infected cells than the quantifications. Furthermore, it seems that in the quantifications the difference is driven by the replicate with the blue dots. Can the authors confirm that they observed a difference in mock versus infected cells when analyzing each experiment separately? Maybe the authors should also consider showing cells with a less prominent effect that is more reflective of the quantification results. Given that the results shown in fig. 4 are central for the conclusions this part should be strengthened.

We thank the reviewer for helping us improve the presentation of the PI4P staining data. In the revised version of Figure 4 (**Figure S5** in the revised version the manuscript), we included a different set of images for PI4P-stained cells, which better reflect the quantification graphs.

In addition, as requested by the reviewer, we analyzed the three independent experiments separately and confirmed that all three show an increase in PI4P levels upon IAV infection, as shown below in **Figure S5B***. Although one of the experiments shows a less pronounced effect compared to the others (n1), the overall trend is consistent across replicates (see below).

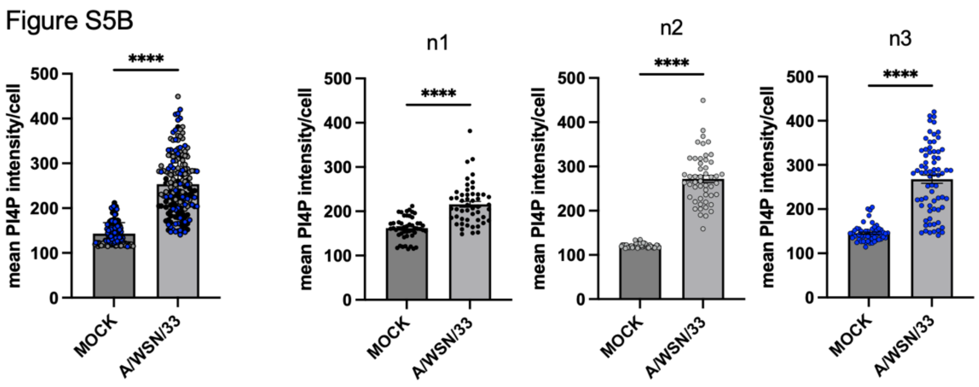


**Figure S5B* (not included in the revised version of the manuscript, related to Figure S5B)**

We did the same kind of analysis regarding the new PI4P-staining data included in Figure 4 of the revised version of the manuscript, i.e. data obtained using with a PI4P-biosensor instead of a PI4P-specific antibody. Again, all three independent experiments reveal an increase in PI4P levels upon IAV infection, as shown below in **Figure 4B***.

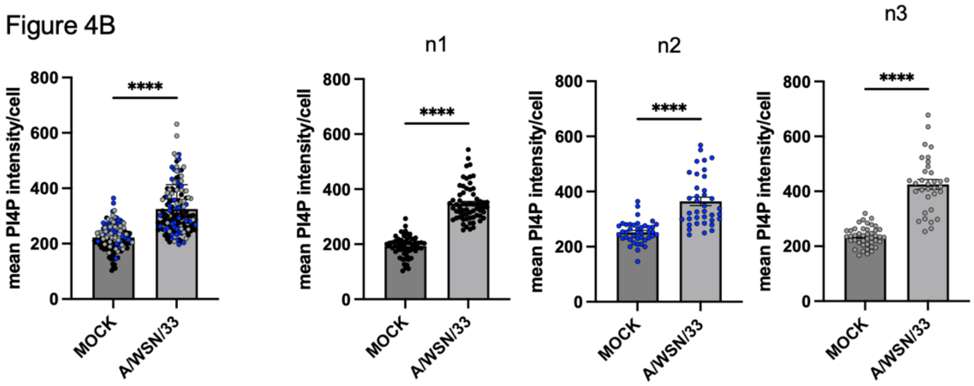


**Figure 4B* (not included in the revised version of the manuscript, related to Figure 4B)**

We believe that these additional data and analyses strongly support our conclusions that IAV infection induces a decrease in PI3P and an increase in PI4P, and we hope they appropriately adress the reviewer's concerns.

**Minor comments:**

- Fig. S4A: The GO terms are difficult to read, and I would appreciate some more annotation. For example, the authors could mark the GO terms that refer to membrane-associated proteins, given that they say these are overrepresented.

We thank the reviewer for pointing to the shortcomings of the Fig S4A.

For clarity, the GO terms that refer to membrane-associated proteins and to intracellular transport/localization/vesicles have been highlighted using blue and green color fonts, respectively.

The Fig S4A legend and main text (page 10 lines 8-9) have been modified accordingly.

- Fig. 3A, S4B: In my opinion, the green and the blue plots next to the volcano plot do not provide or illustrate any information, other than giving the number of proteins found in that category. Either it needs to be explained what the panels add, or they should be deleted.

The green and blue bar plots adjacent to the volcano plots in Fig 3A and S4B are crucial to provide a comprehensive description of the different categories of proteins identified in our proximity labeling experiment. They represent proteins that have quantified intensities from LC-MS/MS data in one condition (Virus or Mock) but not in the other. Since these proteins are « present/absent », no fold-changes or p-values can be obtained, therefore they cannot be visualized in the volcano plot. However, the iBAQ values are strongly correlated with

the concentrations of proteins, therefore they allow to compare protein abundance across conditions even though no fold-change can be calculated.
In summary, while the primary volcano plot provides an overview of the differential protein expression, the green and blue plots are highlighting and ranking key « present/absent » proteins. Notably, the barplots in Fig. 3A and Fig. S4B are highlighting the ATG16L1 and FAM126A proteins of interest, and their abundance with respect to other « present/absent » proteins, which would not be possible if only the volcano plots were shown.

The legends of Figure 3A and S4B have been revised to better explain the information provided in the side plots.

**REVIEWER #3**

This manuscript from Alemany and Da Graça shows a novel role for ATG16L1 and P14P in the influenza infection. This work uses high resolution microscopy to further establish the perturbation of the endoplasmic reticulum upon influenza infection, and a proximity-ligation based proteomics approach to characterize the Rab11A interactome in the presence/absence of IAV. They show that IAV infection results in a change in the relative PI3P/P14P levels, and the P14P punctae colocalize with NP at late stages of infection in a ATG16L1 dependent manner. The work is thoughtfully designed, the writing is clear and the microscopy is striking. The findings will be of broad interest to both influenza virologists and cellular biologists and contains several important observations. The characterization of the Rab11A interactome is thorough (four replicates greatly increase the utility of this dataset) and will be valuable to the field. In addition, the finding that vRNPs make their way to the ER in a Rab11A independent manner is novel and presents a new challenge to understanding the cellular factors involved in IAV trafficking. I have identified a few issues with quantification and MOI, addressing these would strengthen the claims made in the manuscript.

**Major Points**

The data in Figure 6 does not unambiguously show that the effect of ATG16L1 knockdown is occurring at a late stage of viral infection. By performing infections at a very low MOI, multiple cycles of replication are occurring (and the defect in PFU production could theoretically occur at any stage of the life cycle). While the IF data attempts to solve this problem by looking for NP accumulation, not every cell displays a uniform accumulation of NP along the entire cell periphery. The quantification here is hard to fully understand, since cells are classified in a binary fashion (by eye?), and only the percent of cells with any NP at the PM is shown in the graph (ie, only three numbers). My comparison of cells in which I think there is NP at the plasma membrane does not fully agree with the white stars used to denote 'cells with PM NP' in Fig 6E/F so the authors should probably reanalyze this in a way that does not depend on a 'by eye' determination.

We agree with the reviewer that a « by eye » quantification method is subject to bias and not fully satisfactory.

Therefore we re-analyzed our set of images using an unbaised procedure, by using Image J to draw a line along the plasma membrane of all cells present in a given field of vision, and to measure fluorescence intensity along this line. Both ATG16L1 depletion or GSK-A1 treatment significantly reduce the fluorescence intensity signal which supports the notion that they impair the transport of vRNPs to the plasma membrane.

The new quantification data are shown in Figure 6B and 6F of the revised version of the manuscript, and discussed page 15 lines 3-9 and page 16 lines 15-17.

But really, in order to make the claim that this defect in infectious virus production occurs at a late stage in viral infection, the authors should perform synchronized infections at a high MOI, harvest virus after a single cycle of infection, and show that protein production is unaffected by western blot (as in S5D, to rule out a defect in any of the stages prior to assembly/trafficking).

This important point, also raised by Reviewer #1, has been adressed in details above (please see pages 1-3 of the present rebuttal).

We performed infections at a high MOI of 5 PFU/cell. We did not use the standard synchronization protocol, i.e. viral adsorption at +4°C for two hours followed by a shift at 37°C, because such temperature shifts are known to alter membrane trafficking in eukaryotic cells, particularly temperatures around 4°C, that significantly alter various processes, including endocytosis, vesicle transport, and membrane protein localization (Martinez-Alonso et al, Histochemistry and Cell Biology 2007, Breton et al, J Am Soc Nephrol, 1998, Danielsen et al, J Cell

Biol, 1993, Zilberstein et al, Cell, 1980). On the contrary, in all our experiments, we carefully avoided shifts in temperature by systematically using pre-heated medium at 37°C, as indicated in the Materials and Methods section.

I agree, the data shows that the loss of ATG16L1 reduces the number of P14P punctua per cell (based on the quantification, the representative image shown in 5A though looks pretty similar for all three conditions in the P14P channel). However the image shown in 5E does not convincingly make the point that ATG16L changes its localization upon infection- the authors should perform the same rigorous analysis of ATG16L ER-associated punctae as was done in 5C.

We understand the reviewer's request for a rigourous quantification of ATG16L1 association with the ER. The reason why we did not initially perform such a quantification is that ATG16L1 staining is more diffuse than NP or PI4P staining, which makes accurate quantification more challenging.

To accurately quantify ATG16L1 association with the ER, we made use of a machine learning-based method previously described in Da Graça et al. (Life Science Alliance, 2022). To remove fluorescence background before analysis, each channel was converted as a binary mask using the machine learning Ilastik software, then the two masks were merged again, and analyzed for area of codistribution using the color threshold method in Image J. The data confirmed that ATG16L1 association with ER membranes increases upon IAV infection

The new quantification data are shown in **Figure 5F** and referred to page 13 lines 35-36 and page 14 line 1 of the revised version of the manuscript.

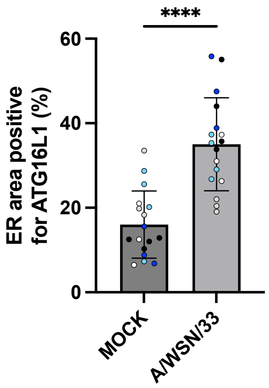


**Figure 5F (revised version).** U2OS-Sec61ß-mEmerald cells treated as in (E) were analyzed with machine learning segmentation and Fiji software to determine the percentage of ER area positive for ATG16L1. Each dot represents the mean of multiple cells from one image, and the data from five independent experiments are shown (white, black, grey and blue and light blue dots). The mean and standard deviation values are represented (17 images/condition). **** : p-value <0.0001, unpaired t-test.

**Minor Points**

The data with ZIKV distracts from the main story (both ER sheets and ER tubules appear to be disrupted, as opposed to just ER sheets for IAV). I would consider removing it.

The data with ZIKV, which show a very distinct pattern of ER remodeling, is an indication that IAV-induced ER membrane alterations are not merely the result of a general stress-response induced by viral infections.

In our view, this observation is of interest, especially in the virology field. Therefore, if the reviewers and editorial board agree, we would prefer to keep this piece of data included as Figure S1B. We propose a rephrasing (page 7 lines 4-8) to better emphasize its worth.

* Figure 1B/2D, the legend mentions black, white and gray dots, but I only see black, gray and blue dots (double check this in all figures that use this schematic).

We apologize for the mistake and have corrected the legend of Figure1B/2D. Indeed, we are systematically using black, gray & blue colors to represent the 3 biological replicates in our datasets.

Figure 2A, I am confused why non-specific Rab11 nuclear staining is enhanced upon Rab11A depletion. It seems plausible to me that this could be bleed through from the NP antibody, and it would help to show the staining for uninfected, depleted cells to demonstrate that the non-specific nuclear staining occurs to the same level in the absence of any NP. The depletion looks convincing by western blot, but this IF image is not as strong an argument for efficient Rab11A depletion.

We thank the reviewer for this comment. To address it, we performed additional RAB11A and NP immunostainings on cells that were treated with Non-Target (NT) or RAB11A siRNAs, and were subsequently mock- or IAV-infected. These control experiments reveal that a nuclear staining with the RAB11-specific antibody is visible in RAB11A-depleted cells, whether they are mock- or IAV-infected cells, i.e. in the absence as well as in the presence of NP staining (Figure S6A, shown below), indicating that is not due to bleed-through from the NP antibody.

Our interpretation is that the nuclear staining corresponds to non-specific background due to cross-reactivity of the RAB11A antibody with some nuclear antigen(s). Two non-exclusive possibilities are that i) the non-specific nuclear staining occurs both in siNT- and siRAB11A-treated cells, and becomes more visible in siRAB11A-treated cells because of a stronger contrast with the negatively stained cytoplasm, and ii) the non-specific nuclear staining is more prominent in siRAB11A-treated cells because of the specific RAB11A antigen is depleted and does not compete with the non-specific nuclear antigens for antibody binding.

These data are shown in **Figure S6A** of the revised version of the manuscript, and are referred to in the legend of Figure 2A.

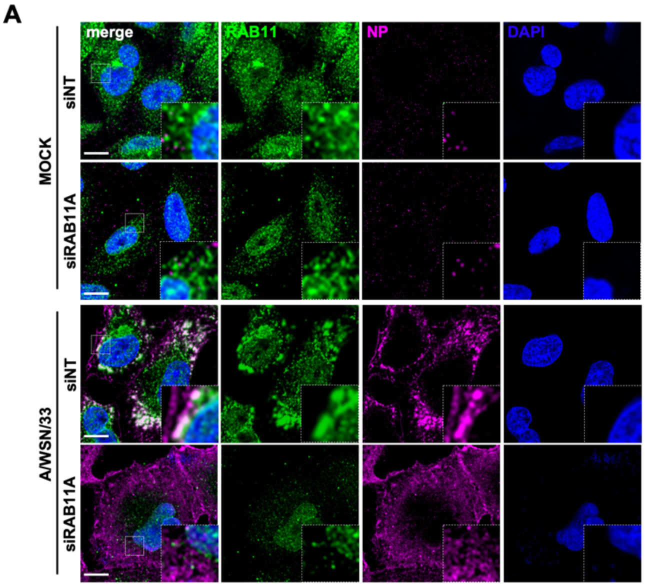


**Figure S6A (revised version)**. A549 cells were treated with RAB11A-specific or control Non-Target (NT) siRNAs for 48 h, and subsequently infected with WSN at a MOI of 5 PFU/cell for 8 hpi or mock-infected. Fixed cells were stained for the viral NP and the cellular RAB11 proteins. Nuclei were stained with DAPI (blue) and cells were imaged with a confocal microscope. Scale bar : 10µm.
